# A study on the prevalence and related factors of frailty and pre-frailty in the older population with diabetes in China: A national cross-sectional study

**DOI:** 10.3389/fpubh.2022.996190

**Published:** 2022-09-23

**Authors:** Xuezhai Zeng, Na Jia, Lingbing Meng, Jing Shi, Yingying Li, Xing Hu, Jiabin Hu, Hongxuan Xu, Jianyi Li, Hui Li, Xin Qi, Hua Wang, Qiuxia Zhang, Juan Li, Deping Liu

**Affiliations:** ^1^Department of Cardiology, Beijing Hospital, National Center of Gerontology, National Health Commission Institute of Geriatric Medicine, Chinese Academy of Medical Sciences, Beijing, China; ^2^Department of Geriatrics, Beijing Hospital, National Center of Gerontology, National Health Commission Institute of Geriatric Medicine, Chinese Academy of Medical Sciences, Beijing, China; ^3^Health Service Department of the Guard Bureau of the Joint Staff Department, Beijing, China; ^4^China Research Center on Aging, Beijing, China; ^5^Institute of Psychology, Chinese Academy of Sciences, Beijing, China

**Keywords:** China, older adults, diabetes, frailty, pre-frailty, related factors, prevalence

## Abstract

**Objective:**

To investigate the prevalence of frailty and pre-frailty and its associated factors in Chinese older adults with diabetes through a nationwide cross-sectional study.

**Research design and methods:**

The data were obtained from the Sample Survey of the Aged Population in Urban and Rural China (SSAPUR), conducted in 2015, which was a cross-sectional study involving a nationally representative sample of older adults aged 60 years or more from 31 provinces, autonomous regions, and municipalities in mainland China. Subjects with diabetes were included in this study. Frailty index (FI), based on 33 potential deficits, was used to categorize individuals as robust, pre-frail, or frail.

**Results:**

A total of 18,010 older adults with diabetes were included in this study. The weighted prevalence of frailty and pre-frailty in older adults with diabetes in China was 22.7% (95% CI 22.1–23.3%) and 58.5% (95% CI 57.8–59.2%), respectively. The prevalence of frailty and pre-frailty among older adults with diabetes from different provinces/municipalities/autonomous regions was significantly different. Multinomial logistic regression analysis showed living alone, poor economic status, ADL disability, and comorbidities were strongly correlated with frailty and pre-frailty in older adults with diabetes.

**Conclusion:**

Frailty and pre-frailty are common in older adults with diabetes in China, and exhibit sociodemographic and geographic differences. In the clinical setting of older adults with diabetes, there is a need to increase awareness of frailty and to advance the early diagnosis and intervention of frailty.

## Introduction

Diabetes is one of the most common chronic non-communicable diseases among the older adults worldwide (1, 2). The 2017 China Diabetes Prevalence Survey showed that the prevalence of diabetes in the older adults aged ≥ 60 years was 30.0%, and China has the largest number of older adults with diabetes in the world (3). Various acute and chronic complications caused by diabetes seriously affect the health status and quality of life of the older adults. Frailty, an emerging global health burden, is a common geriatric syndrome. A meta-analysis of 21 studies from Western high-income countries showed the prevalence of frailty among community-dwelling older adults ranges from 4 to 59%, with an average prevalence of 11% (4), the pooled prevalence of frailty and pre-frailty among community-dwelling older adults in China were 10 and 43%, respectively (5), leading to an increased risk of falls, disability, hospitalization, and death (6–9), as well as an independent predictor of higher healthcare expenditure among older adults (10). As populations age, diabetes and frailty are frequently co-occurring health outcomes, and both frailty and diabetes increase the risk of adverse outcomes in older adults. A meta-analysis showed that older adults with diabetes with frailty had significantly increased risks of hospitalization, disability, and death compared with older adults with diabetes who were not frail (11). Studies have shown that frailty was an important factor affecting the target level of blood glucose management in the older adults with diabetes (12, 13). The International Position Statement on Frailty in Diabetes emphasized that the identification and assessment of frailty should be part of the routine management of people with diabetes (14, 15).

Compared with developed countries, the research on frailty in the older adults in China started relatively recently, there were few comprehensive studies on the prevalence and associated factors of frailty and pre-frailty among older adults with diabetes nationwide. This study used the data of the fourth Sample Survey of the Aged Population in Urban and Rural China (SSAPUR) in 2015 (16), and used the frailty index (FI) model to study the frailty status and related factors of Chinese older adults with diabetes, and to provide evidence for the management of frail older adults with diabetes.

## Methods

### Measurements

#### Participants

**Figure 1:**
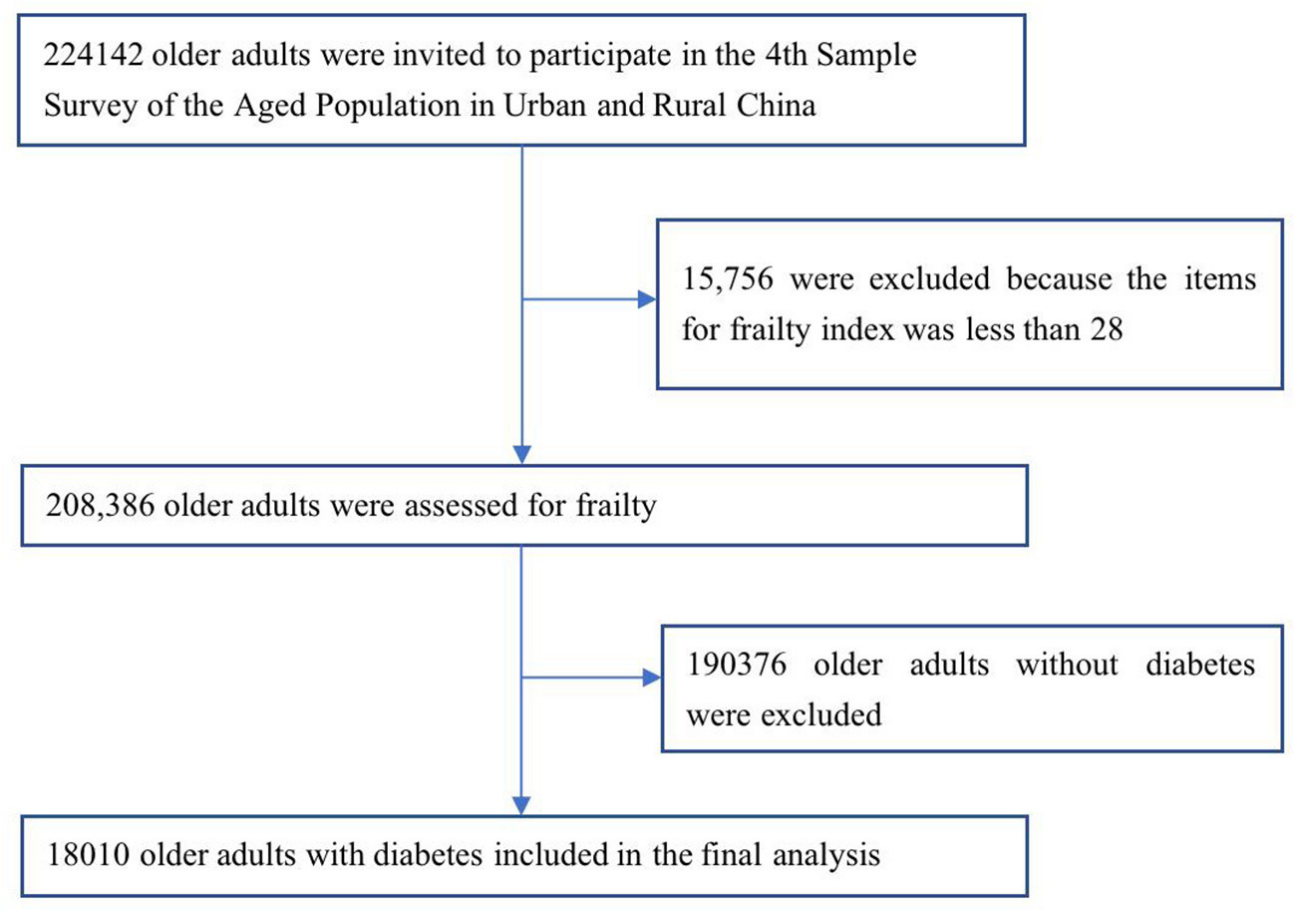
Flowchart of participants sampling of the study on frailty and pre-frailty prevalence in older adults with Diabetes in China.

Data were obtained from the database of the fourth SSAPUR, which was conducted by the China National Committee on Aging from August 1, 2015 to August 31, 2015; this was a national survey of the older population. The participants in the SSAPUR survey were Chinese citizens aged 60 years or more who lived on the Chinese mainland. The survey adopted a sampling design of stratified, multi-stage, probability proportionate to size sampling (PPS) and final stage equal probability (17, 18). The sample obtained was self-weighted to ensure national representation. Sampling took place in four stages, the first stage: The number of samples was allocated according to the proportion of the older population in each province/municipalities/autonomous regions to the whole country. Taking 2,853 districts (counties) in 31 provinces/municipalities/autonomous regions in mainland China as primary sampling units, 466 districts (counties) were selected from them. In the second stage, the total number of older people in each district (county) was used as auxiliary information, and 4 townships (sub-districts) were selected in each district (county) according to the PPS sampling method. The third stage: Using the total number of older people in each townships (sub-districts) as auxiliary information, according to the PPS sampling method, 4 village (residential) committees were selected in each townships (sub-districts). The fourth stage: In the selected townships (sub-districts), according to the list of the older adults reported before the survey, equidistant sampling is adopted, and 30 older people were selected. The design sample size of the survey was 223,680 and the sampling ratio was about one-thousandth. The SSAPUR survey employed questionnaire-based household interviews to collect data. The questionnaire used for the survey was divided into simplified and detailed forms, with 90% of the participants using the simplified form and 10% using the detailed form. If a selected older person declined to accept a visit, died, relocated, could not be contacted (contact at least 3 times), or lived in a long-term older adults' care institution the selected older person would be excluded, a new participant was then selected in order from the candidate list. The survey covered nine aspects, including demographic information, family situation, health status, health care and nursing services, economic status, social activity, living environment, spiritual and cultural life (including psychological status). The research protocol was approved by the National Bureau of Statistics [No. (2014) 87] and the ethics committee of Beijing Hospital (2021BJYYEC-294-01). All participants provided written informed consent before completing the survey. The actual number of collected samples was 224,142. In China, SSAPUR is by far the largest database of older people. A total of 15,756 (7.0%) were excluded because the items for FI was < 28. Among the 208,386 older adults, there were 18,010 older adults with diabetes, determined on the basis of a self-reported history of diagnosis by a physician, were included in this study (Figure 1).

#### Demographics

Demographic characteristics included age (60–64, 65–69, 70–74, 75–79, 80–84, and 85+ years), sex, education (illiterate, non-illiterate), marital status (married, widowed, divorced, and unmarried), ethnicity (Han, non-Han), residency (urban, rural), living status (living alone, not living alone), health checkup within 1 year, hospitalized within 1 year, currently in gainful employment, economic status self-assessed based on categories (very rich, rich, adequate, poor, very poor), convenience of medical cost reimbursement (highly convenient, convenient, less convenient, inconvenient, highly inconvenient), and geographical region (Northeast, Northern, Central, Southwest, South, Northeast, and Southeast).

#### Identification and assignment of health deficits for the FI

The FI items (*n* = 33, each subject had to complete at least 28 of 33 items of the frailty) were selected from the baseline questionnaires of demographic characteristics, physical health, physical functioning, lifestyle, social activity, and mental health status (see Supplementary Table 1), based on Searle's criteria (accumulation with age and no premature saturation) (19). For the binary variables, the presence of a deficit was coded as “1” and its absence was coded as “0”. For variables that included a single intermediate response (e.g., “sometimes” or “maybe”), we used an additional value of “0.5”, and so on. For missing data the FI was calculated based on the items which were present, i.e., the missing variable was excluded from both the numerator and the denominator. The FI was calculated by summing the number of deficits recorded for a patient and dividing this by the total number of possible deficits [the denominator of the FI was adjusted based on the number of questions answered (i.e., 28–33)]. For example, if 30 deficits were considered, and 6 were present in a given person, that person's FI would be 6/30 = 0.2. An FI ≥ 0.25 indicates frailty, an FI < 0.12 indicates robust older adults, an FI 0.12–0.25 indicates pre-frailty.

The FI included eight items (bathing, dressing, toileting, getting in and out of bed, eating, walking around the room, urinary incontinence, and fecal incontinence) of basic activities of daily living (ADL); ten items focusing on chronic diseases included glaucoma/cataract, cardiovascular disease, hypertension, diabetes, gastric disease, bone and joint disease, chronic lung disease, asthma, malignancy, and reproductive system disease; two items focused on feelings of loneliness and happiness; three items focused on geriatric syndrome, including visual impairment, hearing impairment, and history of falls; five items focused on assistive devices (hearing aids, dentures, crutches, wheel-chairs, and adult diapers/nursing pads); three items focused on mobility (needing care from others in daily life, self-rated health status, and exercise); two items focused on social activity (regular leisure activities and regular public service activities).

ADL disability was assessment included six items (bathing, dressing, going to the toilet, getting in and out of bed, eating, and walking around indoors). Each item was assessed using three levels: “can do”, “some difficulty” and “can't do”, and given a score of 0, 0, and 1, respectively; a total ADL score of ≥1 was classified as ADL disability.

### Statistical analysis

SPSS 24.0 software was used for the data analysis. Missing data (5.6% of the participants lacked convenience for medical reimbursement, while educational level was 0.2%, ethnicity was 0.1%, marital status was 1.2%, living alone was 0.1%, financial status was 0.6%, hospitalized within 1 year was 2.0%, medical examination was 1.6%, and medical insurance was 0.2%) were interpolated using the Markov Chain Monte Carlo (MCMC) multiple fill method (20). We calculated prevalence of frailty and pre-frailty in the overall sample and subgroups stratified by sociodemographic, and frailty and pre-frailty weighted prevalence based on the weights created in our study. Continuous variables are reported as the mean ± standard deviation (SD) or as the median and interquartile range (IQR), as appropriate. Comparisons between two or Multivariable groups were made by using a Student's *t*-test or ANOVA for symmetrical continuous distribution, and Mann-Whitney U test or Kruskal-Wallis H test for non-symmetrical continuous distribution. Categorical variables are reported as percentages and were compared using a chi-square test, Bonferroni correction was applied to the results of the two-by-two comparison between groups. Multinomial regression analysis models were used to analyze related factors associated with the prevalence of frailty and pre-frailty in older adults with diabetes.

### Sensitivity analyses

We compared baseline characteristics, the prevalence of frailty and pre-frailty, between older patients with diabetes missing the items for the FI and those without the items missing. Differences were considered statistically significant at *P* < 0.05.

## Results

A total of 224,142 older people aged 60 years or more participated in the Fourth SSAPUR in China in 2015, of whom 15,756 (7.0%) respondents were excluded because their items for FI was < 28. Among 208,386 older adults, the prevalence of frailty was 9.4%, and the prevalence of pre-frailty was 45.8%. Among them, the number of older adults with diabetes were 18,010, and the prevalence of diabetes was 8.6%. The prevalence of diabetes in the frail older adults was 20.5%, the prevalence of diabetes in the pre-frail older adults was 11.0%, and the prevalence of diabetes in the robust older adults was 3.7%.

The average age of older adults with diabetes was 69.7 ± 7.8 years (range 60–105 years); 10,547 (58.6%) were female, average age 70.1 ± 8.1 years, and 7,463 (41.4%) were male, average age 69.4 ± 7.6 years.

The distribution of FI in older adults with diabetes was gamma distributed (statistic = 0.093, *P* < 0.001, see Supplementary Figure 1), ranging from 0.03 to 0.70, with a median of 0.18 (IQR 0.10). The median FI was 0.16 (IQR 0.10) for men and 0.19 (IQR 0.10) for women. The FI value for men was significantly lower than that for women (z = −15.3, *p* < 0.001).

### Prevalence of frailty and pre-frailty amongst older adults with and without diabetes

The prevalence of frailty in the older adults with diabetes was 22.4%, which was higher than that in the older adults without diabetes (8.2%; χ^2^ = 3,884.9, *P* < 0.001). The prevalence of pre-frailty in the older adults with diabetes was 58.5%, which was higher than that in the older adults without diabetes (44.6%; χ^2^ = 1,284.3, *P* < 0.001). The weighted prevalence of frailty and pre-frailty in older adults with diabetes in China was 22.7% (95% CI 22.1–23.3%) and 58.5% (95% CI 57.8–59.2%), respectively, according to the weights created in our study.

### The prevalence of frailty and pre-frailty in older adults with diabetes in different regions of China

**Figure 2:**
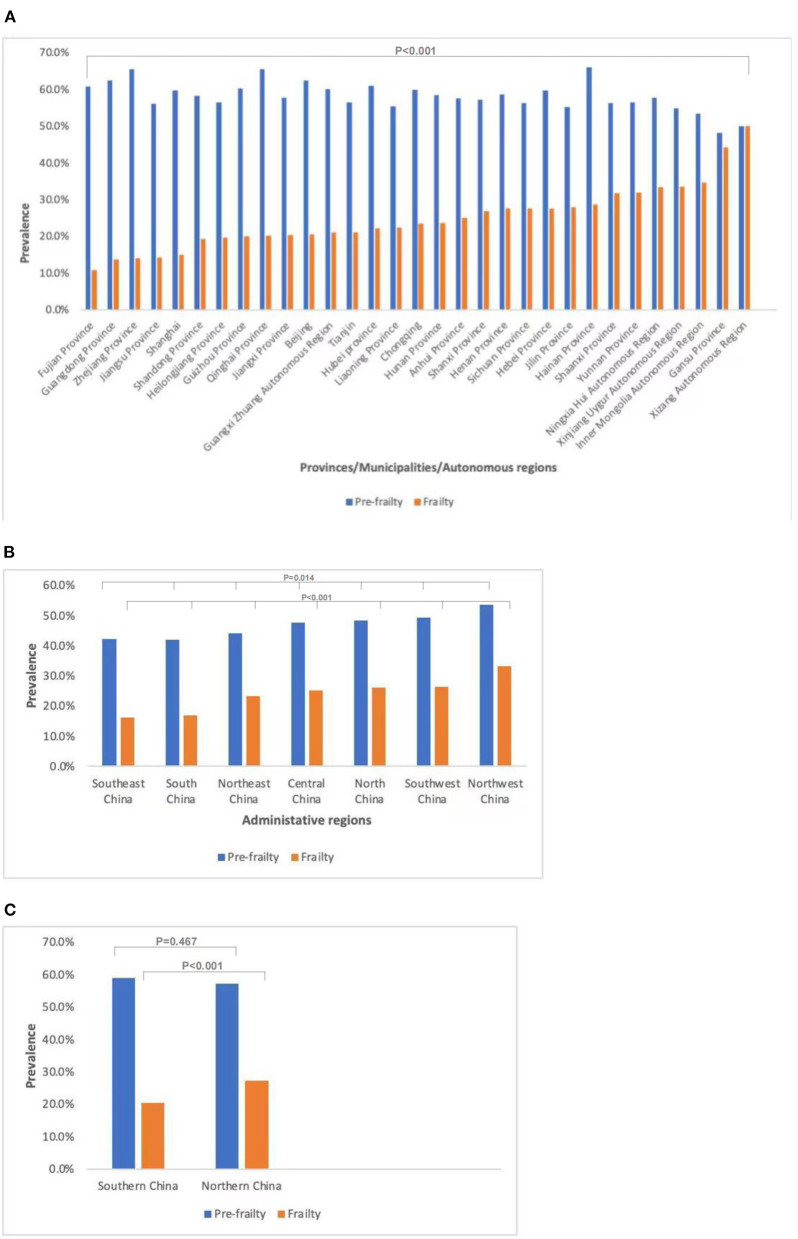
Prevalence of frailty and pre-frailty in older adults with diabetes in different regions of China. **(A)** Prevalence of frailty (*P* < 0.001) and pre-frailty (*P* < 0.001) in older adults with diabetes in different provinces/municipalities/autonomous regions. **(B)** Prevalence of frailty (*P* < 0.001) and pre-frailty (*P* = 0.014) in older adults with diabetes in different administrative regions. **(C)** Prevalence of frailty (*P* < 0.001) and pre-frailty (*P* = 0.467) in older adults with diabetes in southern and northern China.

The prevalence of frailty (χ^2^ = 457.7, *P* < 0.001) and the prevalence of pre-frailty (χ^2^ = 66.2, *P* < 0.001) of older adults with diabetes in different provinces/municipalities/autonomous regions were significantly different. The prevalence of frailty in older adults with diabetes was highest in the Xizang Autonomous Region (50.0%) and lowest in Fujian Province (10.8%); the former was 4.6 times higher than the latter. The prevalence of pre-frailty in older adults with diabetes was highest in Hainan Province (66.1%) and lowest in Jiangsu Province (48.2%); the former was 1.4 times higher than the latter. The prevalence of frailty in different administrative regions was significantly different (χ^2^ = 280.4, *P* < 0.001). The prevalence of frailty was highest in Northwest China (33.2%), followed by Southwest (26.4%) and North China (26.1%); Central China (25.3%) and Northeast China (23.3%) were in the middle, while South China was lower (17.0%), with the lowest in Southeast China (16.1%). The prevalence of pre-frailty in different administrative regions was also significantly different (χ^2^ = 16.0, *P* = 0.014). The prevalence of frailty in northern China was higher than that in southern China (27.2 vs.20.4%, χ^2^ = 99.6, *p* < 0.001), while the prevalence of pre-frailty in northern China was not different from that in southern China (57.3 vs. 59.0%, χ^2^ = 0.5, *p* = 0.467) (Figure 2).

### Prevalence of frailty and pre-frailty in older adults with diabetes

The prevalence of frailty and pre-frailty amongst different groups in older adults with diabetes see Table 1. The prevalence of frailty in older adults with diabetes increased with age. Other factors linked with a higher prevalence of frailty in older adults with diabetes were being female, rural residency, widowed, illiterate, ethnic minority, living alone, hospitalized in the past 1 year, no health checkup in the past year, financial difficulties, inconvenient reimbursement of medical expenses, ADL disability, and combined chronic diseases. There was no difference in the prevalence of frailty and pre-frailty in older adults with diabetes with or without medical insurance.

**Table 1:** Prevalence of frailty and pre-frailty among older adults with diabetes in China in 2015.

	**All**				**Men**				**Women**			
	**Total (*n* = 18,010)**	**Pre-frail**	**Frail**	***P-*value**	**Total (*n* = 7,443)**	**Pre-frail**	**Frail**	***P-*value**	**Total (*n* = 1,0547)**	**Pre-frail**	**Frail**	***P-*value**
Proportional of participants		58.5%	22.4%		100%	58.0%	18.9%		100%	58.9%	24.8%	
Age (years)	69.7 ± 7.8	70.8 ± 7.9	74.6 ± 8.8	< 0.001	69.4 ± 7.6	70.3 ± 7.7	73.6 ± 8.5	< 0.001	70.1 ± 8.1	70.8 ± 8.1	74.3 ± 8.9	< 0.001
**Age group**				< 0.001				< 0.001				< 0.001
60–64	29.4%	58.9%^a^	15.5%^a^		29.7%	56.7%	12.8%^a^		29.2%	60.4%^a^	17.5%^a^	
65–69	25.3%	60.0%^a^	18.8%^b^		25.5%	58.5%	15.6%^a, b^		25.1%	61.0%^a^	21.0%^b^	
70–74	18.4%	60.1%^a^	22.1%^c^		18.5%	59.9%	18.2%^b^		18.3%	60.3%^a^	24.8%^c^	
75–79	14.1%	58.7%^a^	28.6%^d^		13.2%	60.7%	24.6%^c^		14.7%	57.5%^a, b^	31.1%^d^	
80–84	8.7%	53.6%^b^	36.1%^e^		8.6%	55.9%	31.2%^c, d^		8.8%	52.1%^b, c^	39.5%^e^	
≥85	4.1%	49.7%^b^	44.7%^f^		4.5%	52.7%	40.4%^d^		3.8%	47.3%^c^	48.3%^f^	
**Urban or rural area**				< 0.001				< 0.001				< 0.001
Urban	67.3%	58.8%	19.7%^a^		71.2%	57.6%	16.5%^a^		64.6%	59.7%^a^	22.2%^a^	
Rural	32.7%	58.0%	27.8%^b^		28.8%	59.0%	24.7%^b^		35.4%	57.4%^b^	29.6%^b^	
**Education**				< 0.001				< 0.001				< 0.001
Illiterate	25.5%	55.4%^a^	32.6%^a^		8.9%	56.3%	32.0%^a^		37.2%	55.2%^a^	32.7%^a^	
Non-illiterate	74.5%	59.6%^b^	18.9%^b^		91.1%	58.2%	17.6%^b^		62.8%	61.1%^b^	20.2%^b^	
**Marital status**				< 0.001				< 0.001				< 0.001
Married	74.0%	58.6%^a^	18.9%^a^		85.6%	57.5%^a^	17.0%^a^		65.7%	59.6%^a, b^	20.7%^a^	
Widowed	24.5%	57.5%^a^	33.0%^b^		12.0%	58.8%^a^	32.6%^a^		33.5%	57.2%^b^	33.1%^b^	
Divorced	0.9%	74.8%^b^	16.8%^a^		1.0%	76.3%^b^	14.5%^a^		0.7%	73.4%^a^	19.0%^a^	
Unmarried	0.6%	67.6%^a, b^	25.0%^a, b^		1.4%	68.0%^a, b^	24.3%^a, b^		0.1%	60.0%^a, b^	40.0%^a, b^	
**Ethnicity**				< 0.001				0.112				0.001
Han	96.1%	58.6%	22.1%^a^		94.1%	58.0%	18.7%		96.1%	59.0%	24.5%^a^	
Non-Han	3.9%	56.9%	28.2%^b^		5.9%	57.2%	23.1%		3.9%	56.6%	31.9%^b^	
**Living status**				< 0.001				< 0.001				< 0.001
Living alone	12.6%	61.3%^a^	34.1%^a^		8.9%	65.0%^a^	29.0%^a^		15.2%	59.8%	36.2%^a^	
Not living alone	87.4%	58.1%^b^	20.7%^b^		91.1%	57.3%^b^	17.9%^b^		84.8%	58.7%	22.8%^b^	
**Health checkup within 1 year**				< 0.001				< 0.001				< 0.001
No	33.6%	57.2%^a^	25.7%^a^		32.1%	58.0%	21.0%^a^		34.7%	56.6%^a^	28.7%^a^	
Yes	66.4%	59.2%^b^	20.7%^b^		67.9%	58.0%	17.9%^b^		65.3%	60.1%^b^	22.8%^b^	
**Hospitalized within 1 year**				< 0.001				< 0.001				< 0.001
No	61.1%	60.4%^a^	16.4%^a^		62.3%	59.1%^a^	13.3%^a^		60.3%	61.4%^a^	18.6%^a^	
Yes	38.9%	55.5%^b^	31.8%^b^		37.7%	56.2%^b^	28.2%^b^		39.7%	55.0%^b^	34.3%^b^	
**Economic status**				< 0.001				< 0.001				< 0.001
Very rich	1.6%	47.3%^a^	15.5%^a, b^		1.70%	44.0%^a^	14.4%^a, b^		1.5%	50.0%^a, b, c^	16.5%^a, b^	
Rich	16.5%	58.0%^b, c^	14.7%^b^		17.60%	55.9%^a, b^	12.5%^b^		15.7%	59.6%^b, c^	16.5%^b^	
Adequate	60.6%	59.6%^c^	20.7%^a^		60.60%	58.8%^b^	17.5%^a^		60.5%	60.2%^c^	23.0%^a^	
Poor	18.0%	57.6%^b, c^	25.8%^c^		17.00%	58.8%^b^	27.5%^c^		18.8%	56.2%^a, b^	34.8%^c^	
Very poor	3.3%	53.6%^a, b^	41.6%^d^		3.10%	57.2%^a, b^	38.0%^d^		3.5%	51.3%^a^	43.9%^d^	
**Medicare**				0.552				0.371				0.956
Yes	99.1%	58.5%	22.4%		99.2%	57.9%	18.9%		99.1%	58.9%	24.8%	
No	0.9%	61.0%	23.3%		0.8%	65.1%	19.0%		0.9%	58.3%	26.0%	
**Convenience of medical cost reimbursement**				< 0.001				< 0.001				< 0.001
Highly convenient	34.0%	59.2%	19.7%^a^		34.3%	58.0%	16.4%^a^		33.7%	60.1%	22.0%^a^	
Convenient	44.3%	58.0%	23.1%^b^		44.1%	58.1%	19.2%^a, b^		44.4%	58.0%	25.9%^b^	
Less convenient	16.2%	59.4%	23.1%^b^		16.0%	59.3%	20.3%^b^		16.5%	59.5%	25.1%^a, b^	
Inconvenient	3.7%	54.8%	30.5%^c^		3.8%	50.5%	30.3%^c^		3.6%	58.1%	30.7%^b, c^	
Highly inconvenient	1.8%	57.3%	30.2%^c^		1.8%	61.3%	22.6%^a, b, c^		1.8%	54.5%	35.6%^c^	
**Comorbidities**				< 0.001				< 0.001				< 0.001
< 1	11.9%	25.2%^a^	1.8%^a^		14.6%	21.8%^a^	1.3%^a^		10.0%	28.8%^a^	2.3%^a^	
≥1	88.1%	63.0%^b^	25.2%^b^		85.4%	64.2%^b^	21.9%^b^		90,0%	62.2%^b^	27.3%^b^	
**ADL disability**				< 0.001				< 0.001				< 0.001
Yes	6.8%	12.4%^a^	87.6%^a^		3.5%	26.4%^a^	71.5%^a^		4.8%	27.1%^a^	71.6%^a^	
No	93.2%	61.9%^b^	17.6%^b^		96.5%	43.7%^b^	5.4%^b^		95.2%	49.3%^b^	7.9%^b^	

ADL, activities of daily living.

The superscripts a, b, c, d, e, and f indicate the difference in the prevalence of pre-frailty and frailty between the two groups within different subgroups (if the superscript letters are the same between the two groups within a subgroup, the difference between the two groups is not statistically significant. If the superscript letters are different between the two groups within a subgroup, the difference between the two groups is statistically significant), (adjusted *p*-values).

### Multinomial logistic regression analysis of related factors of frailty and pre-frailty in older adults with diabetes

Results on multinomial regressions see Table 2. Taking frailty and pre-frailty as dependent variables and the above factors: age, sex, education, marital status, ethnicity, urban or rural area, living status, health checkup within 1 year, hospitalized within 1 year, economic status, convenience of medical cost reimbursement, comorbidities and ADL disabilities as independent variables, after adjusting for sex, age, urban or rural area, marital status, ethnicity, and education multivariable logistic regression analysis showed that living alone, being hospitalized in the past 1 year, having no physical examination in the past year, very inconvenient medical expense reimbursement, having a difficult economic situation, having ADL disability, and having chronic diseases were factors related to frailty and pre-frailty in older patients with diabetes. Poor economic status, living alone, ADL disability, and comorbidities are factors strongly related to the frailty and pre-frailty of older adults with diabetes.

**Table 2:** Factors associated with frailty and pre-frailty of older adults with diabetes by multinomial logistic regression.

**Variables**		**Pre-frailty vs. Robust**				**Frailty vs. Robust**			
		**OR**	**95% CI**		***p***	**OR**	**95% CI**		***p***
			**Lower**	**Upper**			**Lower**	**Upper**	
Sex	Male	1 (ref)	
	Female	1.148	1.041	1.267	0.006	1.265	1.113	1.437	< 0.001
Age (years)	60–64	1 (ref)	
	65–69	1.197	1.066	1.345	0.002	1.370	1.173	1.601	< 0.001
	70–74	1.349	1.180	1.543	< 0.001	1.654	1.391	1.967	< 0.001
	75–79	1.762	1.495	2.076	< 0.001	2.576	2.108	3.150	< 0.001
	80–84	1.900	1.531	2.358	< 0.001	3.397	2.639	4.373	< 0.001
	≥85	3.177	2.180	4.630	< 0.001	6.594	4.364	9.963	< 0.001
Urban or rural area	Urban	1 (ref)	
	Rural	1.379	1.238	1.537	< 0.001	1.827	1.602	2.083	< 0.001
Marriage	Married	1 (ref)	
	Widowed	1.192	1.023	1.389	0.025	1.272	1.060	1.527	0.010
	Divorced	2.144	1.127	4.078	0.020	1.667	0.771	3.608	0.194
	Unmarried	2.633	1.070	6.482	0.035	2.444	0.891	6.705	0.083
Education	Non-illiterate	1 (ref)	
	Illiterate	1.169	1.027	1.331	0.018	1.551	1.331	1.807	< 0.001
Ethnicity	Han	1 (ref)	
	Others	1.223	0.939	1.593	0.136	1.437	1.052	1.963	0.025
Living alone	No	1 (ref)	
	Yes	5.602	4.310	7.282	< 0.001	9.437	7.092	12.558	< 0.001
Medical checkup within 1 year	Yes	1 (ref)	
	No	1.189	1.075	1.316	0.001	1.382	1.219	1.567	< 0.001
Hospitalized within 1 year	No	1 (ref)	
	Yes	1.402	1.268	1.551	< 0.001	2.634	2.331	2.976	< 0.001
Economic status	Very rich	1 (ref)	
	Rich	1.656	1.212	2.262	0.001	1.222	0.766	1.951	0.400
	Adequate	2.676	1.976	3.624	< 0.001	2.932	1.867	4.606	< 0.001
	Poor	5.569	4.003	7.747	< 0.001	10.230	6.373	16.423	< 0.001
	Very poor	16.181	9.509	27.535	< 0.001	44.957	23.595	85.660	< 0.001
Medical reimbursement	Very convenient	1 (ref)	
	Convenient	1.108	0.999	1.229	0.051	1.357	1.189	1.549	0.018
	Less convenient	1.122	0.974	1.292	0.110	1.309	1.097	1.563	0.003
	Inconvenient	1.258	0.949	1.668	0.110	2.071	1.491	2.876	< 0.001
	Very inconvenient	1.707	1.130	2.579	0.011	2.605	1.617	4.196	< 0.001
Co-morbidities	< 1	1 (ref)	
	≥1	20.413	17.997	23.154	< 0.001	253.339	168.601	380.665	< 0.001
ADL disabilities	No	1 (ref)	
	Yes	224,298,364.801	185,288,984.041	271,520,493.854	< 0.001	5,861,318,550.973	5,861,318,550.973	5,861,318,550.973	< 0.001

ADL, activities of daily living; FI, frailty index; CI, confidence interval; OR, odds ratio.

### Sensitivity analyses

There were no differences in age (69.8 ± 7.3 vs. 69.8 ± 7.4; *p* = 0.705) and sex (male: 42.3 vs. 41.4%; *p* = 0.570) between older diabetic patients (*n* = 926) with missing items (1–5) for the frailty index and those (*n* = 17,084) with no missing items. The prevalence of frailty and pre-frailty did not differ between older diabetic patients with and without the missing frailty index items (24.9 vs. 22.2%; 56.3 vs. 58.6%; both *p* < 0.05).

## Discussion

Our study showed that the self-reported prevalence of diabetes in the older adults was 8.6%, which was consistent with the results of the Chinese Diabetes Surveys. The surveys showed that the prevalence of diabetes among people aged ≥ 60 years was 20.9% in 2013 and 30.0% in 2017, while the awareness rate was about 30.0% (3, 21).

Frailty and diabetes are two important older adults' health problems related to aging. Our study showed that the prevalence of diabetes in the frail older adults was higher than that in the pre-frail older adults, and the prevalence of diabetes in the frail and pre-frail older adults was higher than that in the robust older adults, which was consistent with the results of previous studies (22, 23). Volpato et al. found that frail and pre-frail older adults had a significantly higher risk of new-onset diabetes than robust older adults (22). A meta-analysis showed that the prevalence of diabetes in frail older adults was 34% (23). Sarcopenia in frail older adults might raise blood glucose levels and increase diabetes risk through insulin resistance (24). Muscle weakness has also been shown to be associated with an increased risk of diabetes (25). Higher levels of oxidative stress, elevated levels of inflammatory factors, and endocrine disorders in frail older adults might increase the risk of diabetes (26–28).

Our study showed that the prevalence of frailty and pre-frailty in the older adults with diabetes was higher than that in the older adults without diabetes, which is consistent with previous studies (29, 30). Chhetri et al. found that population with diabetes had a much higher prevalence (19.32%) and incidence (12.32%) of frailty, compared to that of older adults without diabetes (prevalence of 11.92% and incidence of 7.04%) (30). A meta-analysis showed that the pooled prevalence of frailty and pre-frailty in older adults with diabetes was 20.1% (95% CI 16.0–24.2%) and 49.1% (95% CI 45.1–53.1%), respectively (11). A recent meta-analysis reported a median prevalence of frailty of 13% (IQR 9–21) as assessed using frailty phenotypes among older adults with diabetes in the community (31). The above studies confirm that diabetes is a risk factor for the development and progression of frailty. The possible mechanisms included accelerated muscle loss and sarcopenia in diabetes. Hyperglycemia could cause muscle atrophy by inhibiting the growth of skeletal muscle cells, insulin resistance could cause muscle contraction disorders by inhibiting energy metabolism of skeletal muscle cells (32). The complications related to diabetes in the older adults also promoted the occurrence of frailty (24). Studies have shown that for the older adults with diabetes with poor general condition, diet control or taking some hypoglycemic drugs, such as metformin, might increase the risk of malnutrition, leading to an increased risk of frailty (33). In addition, the increased in inflammatory mediators in patients with diabetes (34), and some risk factors of diabetes, such as obesity, could also promote the occurrence and development of frailty (35). A study found people with diabetes or higher HbA1c levels at baseline had a higher frailty level throughout later life, and non-frail patients with diabetes or higher HbA1c also experienced more rapid deterioration of frailty level with aging (36).

Our study further confirmed previously identified factors related to the prevalence of frailty (37–39). Our study found that frail patients with diabetes were older, were more likely to have been hospitalized in the past 1 year, were more likely to have ADL disability, and were more likely to have comorbidities than non-frail patients with diabetes. Our findings are consistent with a study showing that frailty and multimorbidity are common in middle-aged and older adults with type 2 diabetes (40). Our study was a cross-sectional study and was unable to determine the association between ADL disability, chronic disease, and frailty. Studies have shown that people with chronic disease have a significantly higher risk of developing frailty than people without chronic disease (41, 42), and ADL disability was often the result of chronic disease and frailty. Frailty is more reversible than disability, so early detection of frailty in older adults with diabetes is critical. The characteristics of frail older adults with diabetes found in our study suggests that frail older adults with diabetes is a complex and highly vulnerable group. Studies have shown that frail older adults with diabetes are at increased risk of hospitalization, disability, cognitive impairment, reduced quality of life, microvascular and macrovascular complications, hypoglycemia, and death (11, 43). In fact, frailty is an important factor affecting the target level of blood glucose management in the older adults (12, 13). The international position statement on frailty in diabetes emphasized that the identification and assessment of frailty should be part of the daily management of people with diabetes, and recommends a more relaxed glycated hemoglobin target for frail older adults and emphasizes the risk of hypoglycemia (15).

Our study also found that female, rural residency, illiterate, ethnic minority, widowed, living alone, financial difficulties, and very difficult medical reimbursement were associated with the prevalence of frailty and pre-frailty in older adults with diabetes. A longitudinal study showed that unfavorable socioeconomic status in childhood and adolescence might increase the risk of late-life frailty amongst Chinese older adults (44), and another longitudinal study showed that socio-economic status was the factor most closely associated with progression from a healthy state to a more morbid, frail and disabling state (45). Financially well-off older adults had access to better health services, which might help prevent or delay frailty. We did not find any connection between medical insurance and frailty, which was mainly due to the rapid development of China's health system and the establishment of a “universal medical coverage” system. We discovered for the first time that the convenience of medical reimbursement was closely related to frailty in older adults with diabetes. Although basic medical insurance has achieved universal coverage, it is crucial to increase the proportion of medical insurance compensation. The results of our study provided the evidence for the government to formulate corresponding policies: eliminate economic inequality, increase the proportion of medical expenses reimbursement, and improve the level of medical care for vulnerable groups.

Our study found significant regional differences in the prevalence of frailty and pre-frailty in older adults with diabetes. The prevalence of frailty in older adults with diabetes in northern China was higher than that in southern China. China's underdeveloped areas have limited medical resources and lower levels of care for the frail older adults, which at least partially explains these regional differences. The results we have reported provide strong evidence for the need to provide government-funded health resources and services to reduce frailty among older adults with diabetes, to reduce these obvious health service inequalities in rural and underdeveloped areas of China.

Our study has several limitations. First, the data on diabetes were self-reported and might be subject to memory bias. Second, this was a cross-sectional study, so the causal relationship between diabetes and frailty was not analyzed. The effect of frailty on the prognosis of elderly people with diabetes and the glycemic control goals of frail elderly people need further research.

## Conclusion and implications

Frailty and pre-frailty are common among older Chinese adults with diabetes. Frail older adults with diabetes are at increased risk of adverse outcomes. It is necessary to improve the awareness of frailty and promote frailty assessment in the clinical diagnosis and treatment environment of diabetes in older adults. Targeted interventions should be given to frail or pre-frail older adults with diabetes to reduce adverse outcomes and reduce the health care burden.

## Data availability statement

The raw data supporting the conclusions of this article will be made available by the authors, without undue reservation.

## Ethics statement

The studies involving human participants were reviewed and approved by the National Bureau of Statistics (No. [2014] 87) and the Ethics Committee of Beijing Hospital (2021BJYYEC-294-01). The patients/participants provided their written informed consent to participate in this study.

## Author contributions

XZ and NJ wrote the various drafts of the manuscript. XZ, LM, and JS conducted the statistical analyses, with advice from YL, JH, HL, XQ, HW, XH, HX, and JiL were involved in data interpretation. DL, QZ, JuL, and XZ conceived and designed this study. Drafts of the manuscript were revised for important scientific content by XZ, NJ, LM, YL, JH, JS, HL, XQ, HW, XH, HX, JiL, QZ, JuL, and DL. DL is the guarantor of this work and, as such, had full access to all the data in the study and takes responsibility for the integrity of the data and the accuracy of the data analysis. All authors gave final approval of the version to be published.

## Funding

The present study was funded by the National Key R&D Program of China (Grant No. 2020YFC2003000, 2020YFC2003001). The study sponsors were not involved in the design of the study, the collection, analysis, and interpretation of data, writing the report, or the decision to submit the report for publication.

## Conflict of interest

The authors declare that the research was conducted in the absence of any commercial or financial relationships that could be construed as a potential conflict of interest.

## Publisher's note

All claims expressed in this article are solely those of the authors and do not necessarily represent those of their affiliated organizations, or those of the publisher, the editors and the reviewers. Any product that may be evaluated in this article, or claim that may be made by its manufacturer, is not guaranteed or endorsed by the publisher.
