# Supplementary figures and images for: A study on the prevalence and related factors of frailty and pre-frailty in the older population with diabetes in China: A national cross-sectional study
Source: Front Public Health. 2022 Sep 23;10:996190. doi: 10.3389/fpubh.2022.996190 (PMC9539138; doi:10.3389/fpubh.2022.996190)

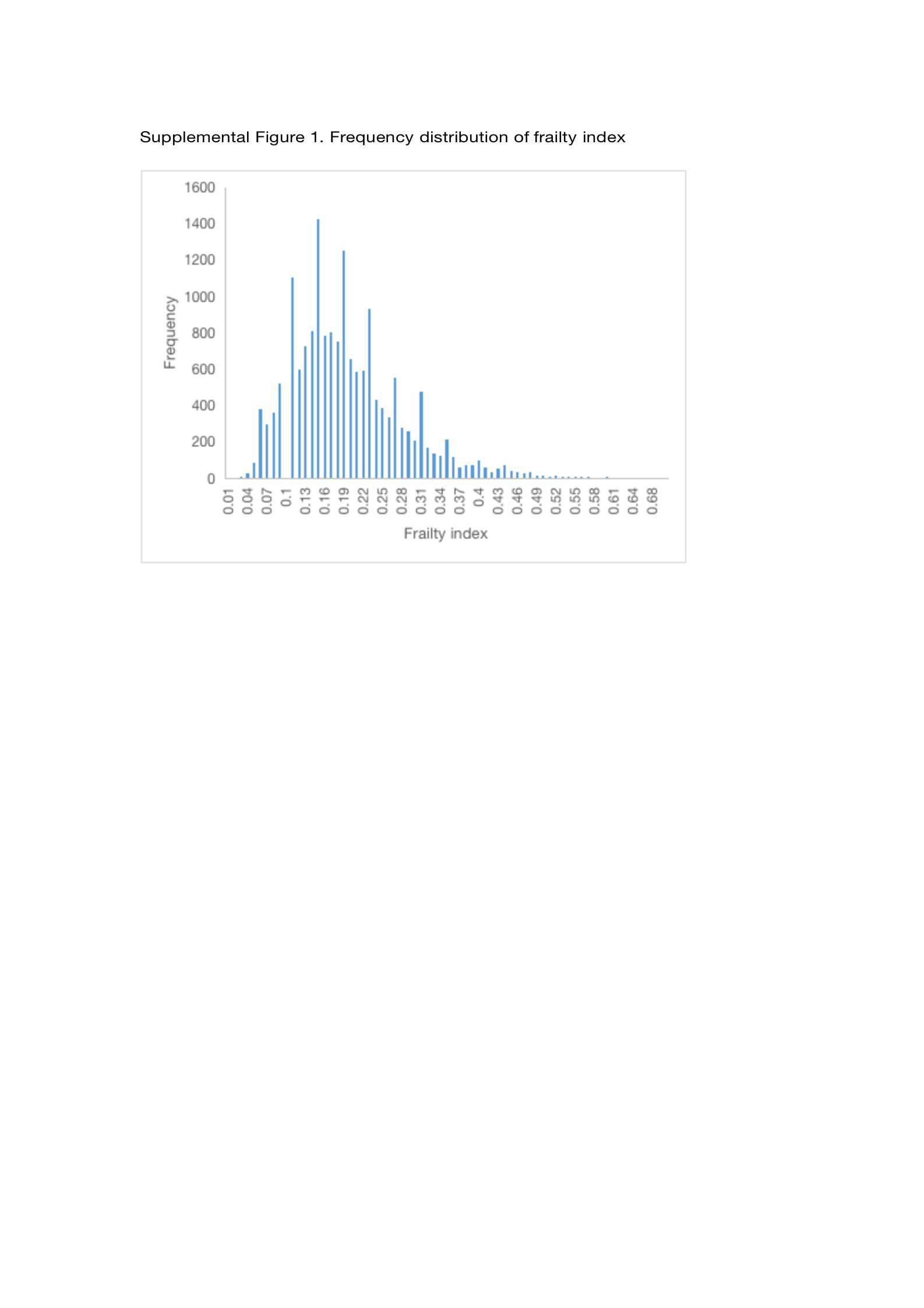

Supplement: Supplementary file 1 [file Image_1.JPEG]
