# Supplementary material for: A study on the prevalence and related factors of frailty and pre-frailty in the older population with diabetes in China: A national cross-sectional study
Source: Front Public Health. 2022 Sep 23;10:996190. doi: 10.3389/fpubh.2022.996190 (PMC9539138; doi:10.3389/fpubh.2022.996190)
Supplement: Supplementary file 2 [file Table_1.docx]

Supplemental Table 1. Variables and calculation of frailty index.

| Frailty index | Cut-off |
| --- | --- |
| **ADL** |  |
| 1. Feeding | Yes =0, with difficulty =0.5, need help =1 |
| 1. Getting dressed | Yes =0, with difficulty =0.5, need help =1 |
| 1. Using the toilet | Yes =0, with difficulty =0.5, need help =1 |
| 1. Getting in and out of bed | Yes =0, with difficulty =0.5, need help =1 |
| 1. Walking indoors | Yes =0, with difficulty =0.5, need help =1 |
| 1. Bathing | Yes =0, with difficulty =0.5, need help =1 |
| **Chronic diseases** |  |
| 1. Glaucoma/cataracts | Yes = 1, no = 0 |
| 1. Hypertension | Yes = 1, no = 0 |
| 1. Diabetes | Yes = 1, no = 0 |
| 1. Cardiovascular diseases | Yes = 1, no = 0 |
| 1. Gastric diseases | Yes = 1, no = 0 |
| 1. Osteoarthritis | Yes = 1, no = 0 |
| 1. Chronic lung disease | Yes = 1, no = 0 |
| 1. Asthma | Yes = 1, no = 0 |
| 1. Cancer | Yes = 1, no = 0 |
| 1. Reproductive system disorders | Yes = 1, no = 0 |
| **Geriatric symptoms** |  |
| 1. Urinary incontinence | Yes = 1, no = 0 |
| 1. Faecal incontinence | Yes = 1, no = 0 |
| 1. Fall history | Yes = 1, no = 0 |
| 1. Visual impairment | Normal = 0, mildly impaired = 0.25, moderately impaired = 0.75, almost blind = 1 |
| 1. Hearing impairment | Normal =0, mildly impaired =0.5, severely impaired =1 3能听清楚 2需要别人提高声音 3能听清楚 |
| 1. How do you feel about your health status? | Excellent =0, good =0.25, fair =0.5, not good =0.75, poor =1 |
| 1. Do you currently need someone to take care of you in your daily life? | Yes = 1,no = 0 |
| 1. Do you feel lonely？ | Often = 1, sometimes = 0.5, never = 0 |
| 1. How happy do you feel? | Very happy = 0, happy = 0.25, moderately happy = 0.5, unhappy = 0.75, very unhappy = 1 |
| **Use of assistive devices** |  |
| 1. Hearing aids | Yes = 1, no = 0 |
| 1. Dentures | Yes = 1, no = 0 |
| 1. Crutches | Yes = 1, no = 0 |
| 1. Wheelchair | Yes = 1, no = 0 |
| 1. Adult diapers/pads | Yes = 1, no = 0 |
| 1. How many times per week do you exercise? | Never = 1, less than once = 0.75, once or twice = 0.5, three-five times = 0.25, more than six times = 0 |
| 1. Do you regularly participate in leisure activities? | Yes = 0, no = 1 |
| 1. Do you regularly participate in public service activities? | Yes = 0, no = 1 |
